# Supplementary material for: Exploratory analysis of the accuracy of age-based maximal heart rate equations across cardiorespiratory fitness levels
Source: PLoS One. 2025 Oct 30;20(10):e0335842. doi: 10.1371/journal.pone.0335842 (PMC12574831; doi:10.1371/journal.pone.0335842)
Supplement: Attachment — Submitted filename: Response To Reviewers.pdf [file pone.0335842.s002.pdf]

GEORGE MASON UNIVERSITY | School of Kinesiology

September 4, 2025

PLOS ONE Editor(s) and reviewers,

We are pleased to re-submit the following manuscript now titled: “Exploratory Analysis of the Accuracy of Age-Based Maximal Heart Rate Equations Across Cardiorespiratory Fitness Levels”. With our resubmission we are providing a revised manuscript with changes noted in track changes and this response letter. Point by point responses to comments are provided below. We would like to express our appreciation to each of the reviewers and the editor for their time.

Reviewer #1

Line 28: the years 2018-2023 are not the same as reported in the method section
Response: Thank you for bringing this to our attention. The years have been corrected. The correct years are 2019-2024.

Lines 71-73: you mention ‘numerous alternative equations’, you need to cite them year, preferably with the name of the equation and/or the author
Response: We appreciate the comment. We’ve added citations to the sentence noted in the review. This sentence is intended to transition to the subsequent paragraph which describes equations in more detail with the equation name.

Lines 88-89: add references for the mentioned studies/formulae
Response: Thank you for the comment. References have been added.

Lines 110-112: add a reference
Response: We have deleted this sentence altogether. Another reviewer suggested that the introduction be shorten and as part of that revision this sentence was decided to be removed.

Line 136: the years 2019-2024 are different to the years in the abstract
Response: The years have been corrected in the abstract.

Discussion: state in the beginning of the discussion whether you can confirm your hypothesis or not.
Response: We appreciate this suggestion. However, in response to another reviewer’s feedback we revised the manuscript to emphasize that this study is exploratory in nature and does not test a priori hypotheses. Instead, we framed our work around the stated objectives, which focus on evaluating the accuracy of multiple MHR prediction equations across CRF levels. Accordingly, the discussion now begins with a summary of the main

findings relative to these objectives, rather than a confirmation or rejection of hypotheses. We believe this aligns with current best practices for reporting exploratory research described by Ditroilo et al.

Ditroilo M, Mesquida C, Abt G, Lakens D. Exploratory research in sport and exercise science: Perceptions, challenges, and recommendations. Journal of Sports Sciences. 2025;43: 1108–1120. doi:10.1080/02640414.2025.2486871

Reviewer #2
This manuscript, titled "Accuracy of Age-Based Maximal Heart Rate Equations in Adults with High and Low Cardiorespiratory Fitness Levels," aims to evaluate the predictive accuracy of seven common age-based MHR equations across different fitness levels. The study addresses an important and clinically relevant question. However, despite its clear structure and thorough data presentation, the work suffers from significant methodological limitations, inconsistent terminology, and analytical choices that reduce its scientific robustness. Below, I outline several major concerns that necessitate a substantial revision of the manuscript.

Abstract:
• Line 21: The acronym "MHR" is used here, while "HRmax" appears elsewhere. Please ensure consistency in terminology throughout the text.
Response: Thank you for bring this to our attention. We have proofread to make sure only MHR is used in the text.

• Line 37: In the abstract results section, p-values are reported. Given that p-values are highly dependent on sample size, it is recommended to also report effect sizes along with their confidence intervals.
Response: We agree with the reviewer's comment regarding the importance of reporting effect sizes alongside p-values. Due to substantial revisions to our analyses, the p-values originally reported in the abstract are no longer present in the revised version. The current abstract highlights variance explained (marginal and conditional R^2 from LME models) and regression coefficients with R^2 values where appropriate, as these provide interpretable indicators of effect magnitude. While traditional standardized effect sizes with confidence intervals were not available for all models (e.g., LME), we made every effort to present complementary measures of model fit and accuracy (e.g., MAE, RMSE, ICC) that contextualize the results beyond statistical significance throughout the manuscript.

Main Text:
• Line 134: It is essential to report the age distribution of the sample. A graphical representation (e.g., kernel density plot) would clarify whether the sample is approximately uniform or biased.
Response: We appreciate the reviewer's suggestion. In the revised manuscript we have added a detailed description of the age distribution of the sample for the overall cohort. To further illustrate the distribution, we have also included a kernel density plot (new Figure 1) that clearly depicts the age distribution across participants. Results of the Shapiro–Wilk test indicated that age was not normally distributed, with the sample showing a greater

proportion of younger participants and a smaller number of older participants (right-skewed distribution). These additions allow readers to both quantitatively and visually assess the distribution of age in the sample.

• Line 137: Sample size is a critical aspect of this type of research. In comparison to the studies cited in the introduction, the sample here appears underpowered. A power analysis is necessary to justify the chosen sample size, specifying the primary outcome and the minimum detectable effect.
Response: We thank the reviewer for this important comment. Because this was a retrospective analysis of graded exercise test data, no a priori power calculation was conducted. Instead, all available data that met inclusion criteria were included. We recognize that this limits our ability to make definitive claims about minimum detectable effects. To address this, we have clarified in the Participants and Design section that the study is exploratory in nature. This framing aligns with recent recommendations for exploratory research in sport and exercise science (Ditroilo et al.), which emphasize transparency and hypothesis-generating insights rather than confirmatory, power-based justifications. We believe this approach is appropriate for the current dataset and clearly communicates the scope and limitations of our findings.

Ditroilo M, Mesquida C, Abt G, Lakens D. Exploratory research in sport and exercise science: Perceptions, challenges, and recommendations. Journal of Sports Sciences. 2025;43: 1108–1120. doi:10.1080/02640414.2025.2486871

• Line 142: Who were the participants? Why were they undergoing GXT testing? What population does this sample represent? These are crucial elements to support the study's external validity.
Response: We thank the reviewer for this important comment. We have revised the Participants and Design section to clarify that participants were individuals from the local community who voluntarily accessed our laboratory's human performance testing services. Most were recreational athletes of varying levels seeking VO_2max and MHR data for training purposes, while others underwent testing for general health assessment. These details have been incorporated to better describe the study population and improve the interpretation of external validity (see Section 2.1).

• Line 169: A scatterplot with age on the x-axis and HRmax on the y-axis should be included, with the regression lines for the seven equations superimposed.
Response: We agree and have added a scatterplot of measured MHR versus age with superimposed regression lines for the seven age-based prediction equations, including sex-specific lines for the Fairbairn model (new Figure 2). This visualization enables direct comparison between observed values and each equation across the age range.

• Line 182: Why was CRF treated as a binary variable? This dichotomization is not appropriate. All analyses involving CRF should be re-run treating it as a continuous covariate. Also, include a kernel density plot of CRF values, as well as a plot of CRF vs age, stratified by sex.
Response: We have re-run all analyses treating CRF (VO_2max) as a continuous covariate rather than a binary classification. In addition, we now include (i) a kernel density plot of

VO_2max to illustrate its distribution (new Figure 1C) and (ii) a scatterplot of VO_2max versus age stratified by sex (new Figure 2). Additionlly, we tested whether the slope of the VO2max vs. age relationship differs for men and women. We found no difference and report this in the results as part of preliminary analyses. These additions address the reviewer's requests and enhance the transparency of the CRF distribution and its relationship with age.

• Line 189: A repeated-measures ANOVA should be conducted using CRF as a covariate, and post hoc tests should compare the regression coefficients (R, a, and b) appropriately.
Response: We thank the reviewer for this suggestion. In the revised manuscript, we used linear mixed-effects models (LMMs) to address the reviewer's concern. Rather than categorizing CRF, we modeled VO_2max as a mean-centered continuous covariate. This approach avoids the information loss associated with grouping or dichotomizing CRF and allows direct estimation of how prediction error changes across the full spectrum of fitness. Because each subject contributed multiple predictions (across seven equations), the independence assumption of ANOVA was not met. By including a random intercept for participant, the LMM explicitly accounts for within-subject correlation, which a standard ANCOVA cannot do without restrictive assumptions. We also included the Equation × VO_2max interaction to test whether CRF modified equation-specific accuracy.

To address the reviewer's request for post hoc tests, we extracted estimated marginal means and simple slopes from the LMMs using the emmeans package, with pairwise contrasts adjusted by the Tukey method. This provides equivalent interpretive value to ANOVA post hoc tests, while accommodating unequal variances, unbalanced data, and random effects.

To further quantify the role of CRF, we fit separate OLS regression models for each equation, regressing absolute error on VO_2max. These models provided slopes (with 95% CI, p-values, R^2, and RMSE) that quantify whether greater CRF systematically increased or decreased error. The plots in Figure 4 illustrate these equation-specific slopes, stratified by sex.

Taken together, the LMMs evaluate the fixed effects of Equation, VO_2max, and their interaction while modeling the correlated data structure, and the regression slopes provide equation-specific estimates of CRF's modifying effect. We believe this approach appropriately addresses the reviewer's concern and strengthens the analysis by including CRF as a continuous covariate but are open to additional feedback to improve the manuscript.

• Line 198: All analyses should be repeated using CRF as a continuous covariate.
Response: Thank you for the feedback. This was addressed as described in a response above.

• Line 236: The dichotomization into "high" and "low" CRF groups is arbitrary and not physiologically grounded. Eliminate this split and instead stratify by sex.

Response: Thank you for the feedback. This was addressed as described in a response above.

• The term “female” should be removed from the table where not necessary.
Response: The term “female” has been removed from the tables. When refering to sex we now more use “men” and “women” throughout.

• Cohen’s d values should be reported as absolute values.
Response: Thank you for the feedback. This has been corrected in the revised manuscript.

• Figure 1: Some predicted MHR values are unreasonably low and likely reflect data entry or estimation errors; these points should be removed. Since all prediction methods are linear equations, distributions should vary only by intercept and scale, not shape. This consistency is not reflected in the figure, indicating possible issues in its construction.
Response: Thank you for bringing this to our attention. Upon further review of the data there were 3 participants whom the integrity of their data appears low based on their age and MHR. We formally now removed outliers using prediction errors greater than 3 standard deviations. This led to the 3 participants being removed and our sample is now 230. We’ve also added figure 2 (MHR vs. age) to illustrate the linear relationship of the prediction equations with age. We appreciate the feedback on this matter.

While the topic of this study is undoubtedly relevant and the manuscript is generally well-written, the current version suffers from multiple critical issues that compromise its scientific validity:
• The sample appears underpowered and lacks justification through a power analysis.
Response: We thank the reviewer for raising these important points. Regarding statistical power, we acknowledge that no a priori power analysis was conducted. Because this was a retrospective analysis of an existing dataset, the sample size was determined by data availability rather than by an a priori calculation. We have clarified this in the revised manuscript (Introduction and Participants sections). We also explicitly note that this work should be considered exploratory in nature, consistent with recent recommendations to distinguish exploratory from confirmatory research. While the sample may be underpowered for certain effects, the purpose of this study is to generate preliminary insights into the accuracy of commonly used MHR equations across different levels of CRF, which can guide future confirmatory work with larger, prospectively designed samples. In addition, we have revised our analytical approach so that CRF is no longer treated categorically. Instead, VO_2max was modeled as a continuous covariate in linear mixed-effects models. This avoids introducing artificial thresholds, reduces loss of statistical power, and allows for more nuanced evaluation of how CRF influences prediction error across the full spectrum of fitness levels.

This being said the reviewer’s points are noted and have been added as potential limitations as well.

We believe these revisions strengthen the methodological rigor and address the reviewer’s concerns about both statistical validity and the treatment of CRF in the analysis.

• CRF is inappropriately treated as a categorical variable, introducing artificial thresholds and reducing statistical power.
Response: We appreciate the comment and have responded to similar comments above.

• Key descriptive data (e.g., age and CRF distributions) are missing.
Response: Thank you for the feedback. These data have been added.

• Graphical representations are either lacking or flawed.
Response: All the figures have been either replaced, updated or removed per the earlier feedback from the reviewer.

• The statistical methods, particularly in regard to interaction effects and covariate inclusion, require complete re-evaluation.
Response: The statistical analysis has been completely redone per the other points of feedback from the reviewer.

Given these major concerns, I do not believe the manuscript is ready for publication in its current form. However, with a thorough methodological revision, clearer data visualization, and a more nuanced statistical approach (especially using CRF as a continuous covariate), the manuscript could be resubmitted and reevaluated. Recommendation: Major Revision
Response: We thank the reviewer for their careful evaluation and constructive feedback. In response, we have thoroughly revised the manuscript to address the methodological, statistical, and visualization concerns. Specifically, we implemented a more nuanced statistical approach by incorporating CRF as a continuous covariate within linear mixed-effects models, which properly accounts for within-subject correlation and avoids the information loss associated with categorizing CRF. To complement these models, we also fit separate regression analyses for each equation to provide equation-specific estimates of the effect of CRF on absolute prediction error. Data visualizations were updated to more clearly depict the influence of CRF and to facilitate comparison across equations and between sexes. We believe these revisions substantially strengthen the methodological rigor and interpretability of the findings, and we appreciate the reviewer's guidance in improving the manuscript.

Reviewer #3
General comments:

In general, the article is well written, and the authors have made an effort to be as precise as possible in respecting the guidelines (e.g., providing information about data access, etc.). However, the study requires improvements in certain aspects of writing and a better justification for the use of specific statistical approaches. Before delving deeper into the specific comments, it is recommended to revise (and add line numbers, as well as after Table 2) the manuscript based on the significant points outlined below.

Title:

As this is a retrospective study, it would be advisable to classify and describe it as exploratory. Including the word exploratory in the title could be helpful. Please refer to the recent article by Ditroilo and colleagues (PMID: 40197233) on the importance of exploratory research (see detailed comments below).
Response: Thank you for the feedback and sharing the article, which was helpful in addressing your comments. We have revised the title to be: *Exploratory Analysis of the Accuracy of Age-Based Maximal Heart Rate Equations Across Cardiorespiratory Fitness Levels*

Abstract:
The abstract, in general, should be reformed once the changes are made in the main text.
Response: Following the substantial revisions to address the comments of the reviewers the abstract has been edited accordingly.

Introduction:
While the Introduction is well written, it is slightly too long. It would be more effective if it were more concise and ideally no longer than 1.5 to 2 pages. Please reduce the content.
Response: We appreciate the feedback and made substantial edits to the introduction. The current length is now closer to 2 pages.

Specific lines comments:
Line 82: The sentence “Furthermore, sex can influence cardiovascular responses to exercise” feels out of context. Consider providing a better transition or justification.
Response: Thank you for the feedback. We’ve edited the paragraph to improve the transition.

Lines 127–128: Avoid stating hypotheses, especially in retrospective studies where a priori hypothesis testing is not feasible and could lead to the so-called HARKing (see Mesquida et al., 2022; PMID: 36533197). Limit this section to stating the main objectives. Again, the article by Ditroilo et al. (PMID: 40197233) may help in distinguishing between exploratory and confirmatory research.
Response: We thank the reviewer for this important comment. In accordance with recommendations on reporting exploratory research (Mesquida et al., 2022; Ditroilo et al., 2025), we removed the hypothesis statement from the introduction and now describe the study objectives as exploratory in nature. We believe this revision appropriately clarifies the scope of the work.

Materials and methods:
The number of the ethical approval should also be present in the main text.
Response: Thank you. This information has been added.

Statistics:
Please specify how data distribution was assessed, and which tests were used for non-normally distributed data, if applicable.
Response: **We thank the reviewer for this comment. In the revised manuscript, we clarified how data distribution was assessed. Specifically, normality of the primary continuous**

variables (age, VO_2max, MHR, and RER) was evaluated using the Shapiro–Wilk test, visual inspection of Q–Q plots, and kernel density plots stratified by sex. This description has been added to the Methods section.

Regarding sample size: since this is a retrospective study, there may be no formal justification. If that is the case, it is still important to disclose this information.
Response: We appreciate the reviewer's comment regarding sample size justification. Because this was a retrospective analysis, no a priori sample size calculation was performed. We have revised the Participants and Design section to clarify that all available data meeting inclusion criteria were analyzed. In line with recent recommendations for exploratory research in sport and exercise science (Ditroilo et al.), we acknowledge that this approach emphasizes transparency and hypothesis-generating insights rather than formal power-based justification.

Please provide a justification for the use of a dichotomous classification of CRF (High vs. Low) based on the 50th percentile. Dichotomizing a continuous variable such as VO_2max may lead to the loss of important information. If possible, consider treating CRF as a continuous variable. On the contrary, if it is not possible for whatever reason, try to justify the decision.
Response: We appreciate the reviewer's insightful comment. After reconsidering our analytic approach, we agree that dichotomizing CRF may obscure important variability in the data. In the revised manuscript, we now treat VO_2max as a continuous variable rather than classifying participants into high vs. low CRF groups. This change preserves the full information available and exploratory examination of how CRF relates to the accuracy of age-based MHR equations.

Specific lines comments:
Lines 150–151: The sentence "After a brief warm-up, the treadmill protocol ramped intensity continuously via speed and/or grade increments every 1–2 minutes" is too vague. Please provide a more detailed description of the protocol.
Response: We appreciate the reviewer's feedback. We have expanded the description of the treadmill protocol to provide detailed information on the warm-up, speed selection, grade progression, speed adjustments, and target test duration to ensure reproducibility.

Lines 180-182: Please, see the comment above on the division between high and low fitness levels.
Response: Thank you for the feedback. The edit has been made.

Lines 209-212: It is not clear how the top and bottom were determined. In particular, a justification of the criteria for this decision should be made.
Response: We appreciate the feedback; however, with the changes to the analysis this has been removed from the manuscript.

Line 211: Use a subscript for VO_2max.
Response: This change has been made throughout.

Results:

After Table 2, line numbers are missing. Please correct this.
Response: This is has been corrected. Thank you.

The choice of the 20th percentile at the top and the bottom remains unjustified and seems arbitrary.
Response: Thank you for bringing this to our attention. With the change to the analysis, namely treating CRF as a continuous rather than dichotomous variable, the analysis of top vs. bottom quintiles has been removed.

Discussion:
Line numbers are missing, making it difficult to reference specific points. Please address this.
Response: This is has been corrected. Thank you.

In general, the discussion should be reframed within the context of exploratory research. For instance, hypotheses should be proposed for future confirmatory studies rather than being inferred from the current findings. Moreover, a part related to the presence of interindividual variability should be incorporated and discussed.
Response: We thank the reviewer for this thoughtful comment. In line with this suggestion, the discussion has been revised to explicitly frame the work as exploratory and to avoid inferring hypotheses from the present findings. Instead, we now highlight areas where future confirmatory studies could test specific hypotheses. We also incorporated interindividual variability throughout the discussion and the first paragraph of the practical implications is now:

One of the clearest implications of this study is the considerable individual variability in MHR, even after accounting for age, sex, and CRF. The 95% limits of agreement spanned approximately ±20 bpm for all formulas. In practical terms, this degree of error could shift an individual across two heart rate training zones (e.g., from moderate to vigorous intensity), which has meaningful consequences for exercise prescription. Thus, while age-based MHR prediction equations are useful for providing population-level estimates and general fitness guidance (e.g., prescribing approximate target zones in a group class), they are not sufficiently precise for individualized programming, athletic performance optimization, or clinical decision-making. In these contexts, reliance on prediction equations could lead to systematic under- or over-prescription of training intensities, underscoring the need for direct measurement of HR_max when accuracy is critical.

• From the sentence “This suggests that although individuals with lower CRF may exhibit slightly smaller prediction errors...” to the end of the paragraph should be revised in light of the exploratory nature of the study.
Response: We appreciate your feedback and agree with the point. However, with the substantial revisions this sentence is no longer included in the discussion.

• Figure 3 should be moved from the discussion to the results section.
Response: Figure 3 has been redone based on the new analyses and moved to the results section as Figure 6 now.

• The sentence “Although there were no statistically significant differences in prediction accuracy between High and Low CRF individuals in the primary analysis, the descriptive analyses revealed that individuals with higher CRF may exhibit greater prediction error across most equations” is particularly important. It is commendable that non-significant results are reported and discussed as did in the present study! Is is also possible to push this aspect in your discussion.
Response: We thank the reviewer for this thoughtful and encouraging comment. We agree that transparently reporting and discussing non-significant findings is important. In the revised manuscript, this specific sentence has been removed due to the updated analytical approach; however, we sincerely appreciate the reviewer’s recognition of this effort.

• The statement “Exercise professionals should consider additional methods, such as perceived exertion or heart rate monitoring over time with wearables” is valid and could be expanded. Consider referencing Teso et al. (2022; PMID: 34967799) and Ferri Marini et al. (2024; PMID: 38711624) for additional context on heart rate prescription limitations and the relationship between RPE and physiological measures.
Response: Thank you for this helpful suggestion. We expanded the discussion to emphasize the limitations of heart rate–based prescription and the complementary role of RPE and wearable monitoring, citing Teso et al. (2022) and Ferri Marini et al. (2024) as recommended.

• The sentence “Specifically, rather than offering a single-point estimate (e.g., “Your MHR is 180 bpm”), professionals can provide prediction intervals (e.g., “Your MHR is likely between 175–185 bpm”) to aid their clients’ understanding of the errors associated with MHR estimation.” sounds colloquial, it can be reformed in a more scientific way.
Response: We have revised the sentence to adopt a more scientific tone by reframing the example of prediction intervals as a methodological recommendation rather than a colloquial illustration. The paragraph now emphasizes prediction intervals as a transparent way to convey estimation uncertainty.

• The sentence “Alternatively, when precision is critical, such as in clinical exercise testing or high-performance athletics, direct MHR measurement via graded exercise testing remains the gold standard” is debatable. In high-performance settings, maximal testing may not be feasible due to potential training disruption. Consider revising or removing this statement.
Response: We appreciate this comment and understand that the statement may be debatable. To avoid overgeneralization, we have removed the sentence regarding graded exercise testing as the gold standard in high-performance settings.

Conclusion:
It is important to state in the conclusion that all the findings of the present study should be confirmed in future confirmatory studies.
Response: We appreciate this suggestion. In line with the reviewer’s feedback, we revised the conclusion to explicitly acknowledge the exploratory nature of the study and the need for future confirmatory research. The final sentence of the conclusion now reads:

"***Importantly, the present results should be viewed as exploratory and confirmed in future confirmatory studies*.**"

Figures:
Please, check all the figure titles and labels. For instance, Figure 2 (a, b, c, etc.) contains a spelling error in the left panel (i.e., Cardorespiratory fitness instead of Cardiorespiratory). Still, in the figures, although they appear clear and sharp when downloaded, they are somewhat blurred in the PDF file. Verify that they are meeting the minimum quality requirements and, if necessary, adjust them accordingly.
Response: Thank you for the feedback. With the new analyses the figures have been redone, with several added due to suggestions from another reviewer. We agree that the image quality is not high in the PDF but when downloaded are acceptable. Upon checking the quality requirements are met but unfortunately are blurring in the proof that is produced from the online system.

Data availability:
Even if the data are uploaded correctly to OSF, it is still necessary to cite them in the main text. Furthermore, it is probably better to anonymize the data.
Response: Thank you for bringing this to our attention. We have anonymized the data on OSF and provided a link to the data in the statistical analyses section of the methods.

Regards,

Joel Martin, PhD
George Mason University
Associate Professor, Kinesiology
