# Supplementary material for: Exploratory analysis of the accuracy of age-based maximal heart rate equations across cardiorespiratory fitness levels
Source: PLoS One. 2025 Oct 30;20(10):e0335842. doi: 10.1371/journal.pone.0335842 (PMC12574831; doi:10.1371/journal.pone.0335842)
Supplement: Attachment — Submitted filename: Response To Reviewers R2.pdf [file pone.0335842.s003.pdf]

GEORGE MASON UNIVERSITY | School of Kinesiology

October 10, 2025

PLOS ONE Editor(s) and reviewers,

We are pleased to re-submit the following manuscript: "Exploratory Analysis of the Accuracy of Age-Based Maximal Heart Rate Equations Across Cardiorespiratory Fitness Levels". With our second resubmission we are providing a revised manuscript with changes noted in track changes and this response letter. Point by point responses to comments are provided below. We would like to express our appreciation to each of the reviewers and the editor for their time.

Reviewer #1
The authors have addressed all comments. No further changes are needed. The submission is ready to be accepted for publication
Response: Thank you for your positive feedback and recommendation. We appreciate your time and support throughout the review process.

Reviewer #2
Overall, the authors have made a commendable effort to improve the manuscript, implementing appropriate modifications where necessary. Below are some specific comments that may help further enhance the quality and readability of the work.
Response: Thank you for your positive feedback and recommendation. We are especially grateful for your thoughtful suggestions during the first round of review, which helped us significantly improve the quality of the paper. We appreciate your time and support throughout the process.

Specific comments:
Line 65. The sentence "which can be impractical and/or risky for many" can be improved and given in a more scientific language.
Response: Thank you for the feedback. We agree that the writing was colloquial and have revised to better reflect scientific language and improve clarity.

Line 82. The sentence "sex can also influence cardiovascular responses to exercise", while can be generally understood, contains a fundamental pitfall. Indeed, it is common for researchers to refer to sex as something that can influence responses to exercise, but this interpretation seems to suggest a reverse causality. This sentence should be then rephrased giving something like sexes can respond differently to exercise.
Response: Thank you for pointing out this important nuance. We agree that the original phrasing may have unintentionally suggested reverse causality. We have revised the sentence to: "While these MHR prediction equations rely primarily on age, cardiovascular

responses to exercise may differ between sexes due to physiological and hormonal differences [12],” to more accurately reflect the relationship and improve scientific clarity.

Line 106. It could be avoided to define age as a “dominant predictor” if considering that usually the predictor is defined as something that shows whether something is likely to happen.
Response: Thank you for pointing this out. We agree that calling age a “dominant predictor” could imply a causal or probabilistic role beyond what our data and prior literature support. Our intent was to note that age is typically the most informative single covariate in population equations for MHR, not to imply determinism.

Lines 137-139. The sample size part is now well explained, the recommendation here is also to cite the article from Lakens (https://doi.org/10.1525/collabra.33267) on the sample size justification and adapt the sentence consequently.
Response: We appreciate the suggestion to cite Lakens. We revised the Methods to (i) cite Lakens’ framework on sample-size justification and (ii) explicitly justify our approach for a retrospective exploratory analysis. We also clarify that future confirmatory work should plan sample sizes for desired precision or a smallest effect size of interest.

Lines 195-196. For this procedure does not seem an arbitrary decision, please provide a citation here.
Response: Thank you for the comment. We added some text to explain the decision for the outlier detection procedure.

Line 218. There is one more space after “interaction”, please remove it and check all over the text if this happened again.
Response: Thank you for the edit. We fixed in this line and proofread the rest of the document to correct any other instances.

GXT testing protocol. Then, all the participants experienced (about) the same starting speed and the same increments? If this is the case, was the time to task failure between 8 and 12 for every participant? Moreover, how running in a 10% slope could have influenced the responses to exercise? Being a not common way of running it could be expected that people were not able to reach their maximum effort because of leg pain or other peripheral discomfort. The impact should be at least acknowledged.
Response: Thank you for this helpful comment. In our dataset, time to task failure was approximately 8–12 minutes for the majority of tests (~≥80%), although exact durations were not available for all records. Starting speed varied by running ability, and it was uncommon for participants to reach the 10% grade. We agree that running at a 10% slope can increase local muscular demand and may prompt earlier termination due to peripheral discomfort rather than cardiorespiratory limitation. We have clarified these points in the Methods (individualized starting speed, grade-then-speed progression, 8–12-minute target) and added a Limitation noting that use of a 10% grade could, in some cases, constrain attainment of true maximal effort and thereby slightly underestimate MHR in a subset of participants.

Table 1. This table can be improved visually. Indeed, it is advisable not to use all the borders but

just the one at the top (i.e., below headings) and one at the bottom (i.e., between the last row and the notes). Additionally, it is suggested to use males and females instead of men and women, both in this table and all over the text.
Response: Thank you for the feedback. The changes have been made to tables 1 and 3.

Table 2 is formatted as suggested for Table 1. It is advisable to be consistent across all the tables and adjust also Table 3 etc.
Response: Thank you again for the feedback. We have revised tables 1 and 3 accordingly.

Lines 412-413. Please, use males and females instead of men and women.
Response: We appreciate the reviewer bringing this to our attention. The edit has been made throughout the manuscript.

Discussion. While the range of age is quite wide in the present study, there is no mention of it as a possible limitation. It should be included, at least discussed as the age ranges also outside the usual definition of adults (e.g., also people above 65 yrs old). How could it have an impact on the results?
Response: Thank you for this suggestion. We added a limitation noting that our broad adult age range (18–68 y, including a subset >65 y) may introduce heterogeneity via age-related medications and chronotropic changes that could attenuate associations.

Line 425. Please, modify men and women also in the discussion.
Response: We appreciate the reviewer bringing this to our attention. The edit has been made throughout the manuscript.

Line 444. The sentence "may exhibit more stereotypical physiological responses to maximal exertion" should be improved and explained more to allow the reader to understand the meaning of this paragraph.
Response: Thank you for this suggestion. We revised the sentence to avoid the vague term "stereotypical" and to explain the proposed mechanism more explicitly.

Lines 493-495. These lines are correct and explain a good point. The only suggestion here could be to explain just in a few more lines why HR has limitations (i.e., the presence of a cardiovascular drift).
Response: Thank you for the suggestion. We added a sentence explaining why HR can be limited for within-session prescription, emphasizing cardiovascular drift and lag as common challenges.

Lines 495-497. In this line there is a great point. For instance, regarding safety, there always should be awareness on the fact that in certain situations where HR cannot be used or can be a misleading parameter (e.g., patients under beta-blockers medications) require attention to different parameters for training monitoring. To be general, you can also expand this section using references of papers that tried to prescribe and monitor aerobic exercise intensity in treadmill running using different approaches and parameters, such as the papers in both general and clinical populations from Faricier et al (PMID: 38980336 and PMID: 39999365) and the papers from Nuuttila et al (PMID: 38956784 and PMID: 35975912). These references and topics

could add great value to this piece of discussion.
Response: Thank you. We expanded the final sentences plus cited examples of alternative/intensified monitoring and prescription approaches in both general and clinical populations. We appreciate the Faricier et al and Nuuttila et al. articles shared.

Lines 515-516. The sentence "As such, the study may be underpowered to detect small effects." is just partially correct. Indeed, medium effects can be difficult to get with an underpowered design. Furthermore, underpowered design can also lead to an increase of the proportion of false positives in a literature where publication bias is present. It is advisable to include both in the text. For a reference on that, please check the article from Mesquida et al. (PMID: 36533197), in particular in section 2.2.2 of the paper (i.e., "Consequences of underpowered designs").
Response: Thank you for this suggestion. We now clarify that limited power can impede detection of small to medium effects and, in literatures with publication bias, may inflate the proportion of false positives. We added Mesquida et al. as a reference and updated the Limitations accordingly.

Lines 531-532. The sentence "who may systematically deviate from general trends." Should be expanded in the view of making readers understand what is meant.
Response: Thank you. We expanded the sentence to specify how these subgroups may differ (medications, chronotropic changes, training adaptations), clarifying why individual prediction error can be systematically biased in these populations.

Lines 536-538. The sentence "The results illustrate both the utility and the limitations of age-based MHR equations—useful for population-level estimates, yet insufficiently precise for individualized programming." is to some extent counterintuitive. Indeed, it should be better explained why something could show both utility and limitations. Probably this sentence is more suitable for a discussion section, instead of a conclusion.
Response: Thank you for noting the potential ambiguity. We revised the language to explicitly state when age-based MHR equations are useful (group-level/initial targets) and when they are limited (individualized programming given typical ±7–10 bpm error and heterogeneity).

Figures. Please, as already asked for in the text, make sure that in all figures men and women are changed with males and females.
Response: This change has been made to all figures.

Figures 5 A, B, C, D, E, F, G. Please, try to avoid that the mean bias is overlapped with the plot dots.
Response: Thank you for this suggestion. We have moved the mean bias label to the top of each plot.

OSF raw data file. Please, consider modifying the column name which refers to gender. Indeed, it should not be confused with sex, which is more related to what has been made in the present article (i.e., it was considered as a biological factor).
Response: We appreciate this feedback and have modified the column name.

Regards,

Joel Martin, PhD
George Mason University
Associate Professor, Kinesiology
